# Opportunities and challenges for people-centered multi-hazard early warning systems: Perspectives from the Global South

**DOI:** 10.1016/j.isci.2025.112353

**Published:** 2025-04-04

**Authors:** Mirianna Budimir, Robert Šakić Trogrlić, Cinthia Almeida, Miguel Arestegui, Orlando Chuquisengo Vásquez, Abel Cisneros, Monica Cuba Iriarte, Adama Dia, Leon Lizon, Giorgio Madueño, Alioune Ndiaye, Miluska Ordoñez Caldas, Tamanna Rahman, Bikram RanaTharu, Alpha Sall, Dharam Uprety, Chris Anderson, Colin McQuistan

**Affiliations:** 1 Practical Action, Rugby CV21 2SD, UK; 2 International Institute for Applied Systems Analysis (IIASA), Laxenburg 2361, LA, Austria; 3 Practical Action, Lima 15704, Peru; 4 Practical Action, La Paz 2922, Bolivia; 5 Practical Action, Dakar, Senegal; 6 Practical Action, Dhaka 1205, Bangladesh; 7 Practical Action, Kathmandu 15135, Nepal

**Keywords:** Earth sciences, Human geography, Social sciences

## Abstract

This perspective critically examines the challenges and opportunities of implementing people-centered multi-hazard early warning systems (MHEWS) in the Global South. Despite global initiatives, such as the Early Warnings for All initiative, operational realities lag behind. By exploring the needs of the most vulnerable and how core concepts of multi-hazard thinking (e.g., hazard interrelationships and vulnerability dynamics) integrate into different pillars and cross-cutting components of an MHEWS, the perspective highlights a mismatch between current ambitions and realities on the ground. Drawing on extensive experience from Practical Action, we identify opportunities to move toward MHEWS through outlining potential entry points in research, policy, and practice. We emphasize a need for localized, inclusive strategies that genuinely address the needs of the most vulnerable populations and fully encompass the meaning of multi-hazards, including hazard interrelationships, the dynamics of risk components, and the complexity of multi-hazard impacts.

## Graphical abstract

**Figure:**
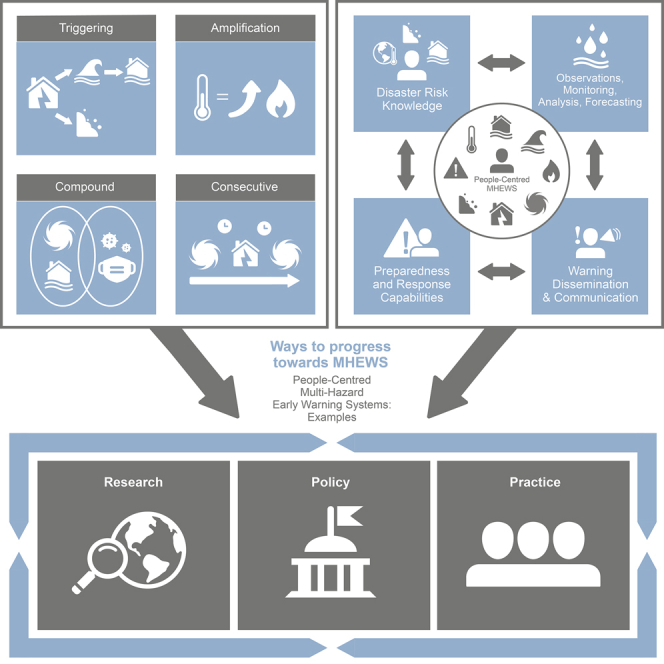

## Introduction

In 2022, the Global Risk Assessment Report by the United Nations Office for Disaster Risk Reduction (UNDRR) highlighted that, despite progress, risk creation is surpassing risk reduction, leading to more disasters, economic losses, and increased vulnerabilities such as poverty and inequality.^1^ We are in an era of escalating and complex climate risks,^2^ with countries in the Global South disproportionately affected,^3^^,^^4^ despite contributing minimally to the problem’s origin. To effectively reduce current and future disaster risks, various approaches are available at individual, community, city, regional, and national levels, guided by global frameworks like the Sendai Framework for Disaster Risk Reduction 2015–2030.

This paper focuses on early warning systems (EWSs), regarded as a key strategy for risk reduction and resilience building, as they enhance understanding of natural hazards, provide timely warnings, and allow for early action to prevent avoidable consequences.^5^ EWSs are defined as ‘integrated systems of hazard monitoring, forecasting and prediction, disaster risk assessment, communication, and preparedness activities that enable timely action to reduce disaster risks before hazardous events’.^6^ EWSs are widely recognized as effective and feasible measures for risk reduction, saving lives and livelihoods and providing at least a 10-fold return on investment.^7^ For instance, a study by Pappenberger et al.^8^ found a potential monetary benefit of cross-border and intercontinental European floods EWS to be of the order of 400 Euros for every 1 Euro invested. EWSs operate across various administrative levels—local, municipal, national, regional, and global. While a holistic, cross-boundary, and multi-actor approach is ideal (including both state and non-state actors^9^), EWSs are typically designed in a top-down manner. National agencies are often tasked with specific roles: hazard data collection (e.g., geological services for landslides), alert issuance (e.g., National Hydro-Met Services), and emergency response (e.g., Disaster Risk or Emergency Management Agencies).

Traditionally, EWSs were designed for single hazards, but there is growing interest in multi-hazard early warning systems (MHEWS). For instance, the Sendai Framework for Disaster Risk Reduction 2015–2030, where Target (g) urges countries to ‘substantially increase the availability of and access to multi-hazard early warning systems’.^10^ In 2022, the UN launched the Early Warnings for All (EW4All) initiative, aiming to protect everyone with an MHEWS by 2027.^7^ The World Meteorological Organization (WMO) has also issued guidelines for multi-hazard impact-based forecasting,^11^ and interest in multi-hazard risk management is rising in the anticipatory action community.^12^ This shift aligns with the broader move from single- to multi-hazard risk management in science, policy, and practice.^13^

What are MHEWS, why are they needed, and how do they differ from traditional EWSs? To answer this, one must first understand the term “multi-hazard”, which refers to multiple hazards affecting a region and their interrelationships, such as triggering, amplifying, or consecutive events.^6^ This involves not only understanding individual hazards (e.g., heatwaves and floods) but also their interactions (e.g., earthquakes triggering landslides). Risks from interrelated hazards are often recognized as greater than those from individual hazards.^13^^,^^14^^,^^15^ Ignoring these interactions can make warnings dangerous. For example, during co-occurring tornadoes and flash floods in the United States, contradictory advice was given: seek low ground for tornadoes and higher ground for floods.^16^ At its core, MHEWS aim to address the complexities of multiple hazards and their interrelated effects. According to the UNDRR, MHEWS are designed to manage hazards that may occur independently, simultaneously, in sequence, or cumulatively over time.^6^ Despite the global push for MHEWS, operational realities differ significantly. The 2023 report on the Global Status of Multi-Hazard Early Warning Systems^17^ states that only 52% of the world is covered by an EWS, with considerable regional disparities: only 46% of Least Developed Countries and 39% of Small Island Developing States are covered by an MHEWS. There is also no clear analysis of whether these MHEWS cover multiple single hazards or consider interrelated hazards and impacts across the EWS chain. Even the latest Words into Action Guide to MHEWS^18^ gives limited attention to how interconnected hazards and risks might be addressed through an MHEWS. Research indicates that few examples of operational MHEWS exist in humanitarian settings, and the current ambitions for MHEWS are not reflected in practice.^12^^,^^19^^,^^20^ Thus, the term MHEWS has often become synonymous with EWS, more indicative of ambitions than actual implementation.

In this perspective, we examine the challenges, opportunities, and realities of developing MHEWS that address both single and interrelated hazards, with a focus on the unique circumstances of Global South countries. We also identify potential entry points to work toward an MHEWS by focusing on concrete actions in research, policy, and practice. The aim of this perspective is not to provide a systematic review of the literature on this topic, but rather to provide a view shaped by experiences of an international development NGO, Practical Action, with decades of experience working on people-centered EWS in Latin America, Africa, and Asia. A short description of Practical Action and its history of work in EWS is provided in Box 1.

Box 1 **Practical Action’s work on EWS**

Practical Action (PA) is an international development organization working with communities living in areas prone to extreme climate hazards across Latin America, Africa, and Asia. PA is striving to develop and improve EWS in their country programs (Peru, Bolivia, Nepal, India, Bangladesh, Senegal, Malawi, and Zimbabwe) as well as taking the lessons learned through technical assistance to other Global South contexts for over two decades. In all of PA’s work, the aim is that those most at risk are prepared so that weather events do not become disasters. The organization takes a systems approach by working across all components of the EWS, connecting and embedding local needs and capacities with government-led municipal or national agencies and systems. Their work bridges research, practice, and policy sectors with a focus on driving inclusive, equitable, and sustainable outcomes. The work has evolved over time but has always taken a holistic perspective and placed the diversity of the most at-risk people at the center. In addition, PA has collected evidence to support their global level advocacy activities sharing learning on effective, people-centered EWS beyond the communities they work with directly.

We recognize the significant opportunity created by the EW4All initiative to rethink what MHEWS truly means and how to design systems that work across settings, particularly for the most vulnerable. Furthermore, we see a potential in the EW4All initiative to engage with overcoming some of the disadvantages, limitations, and gaps of EWS, including false warnings,^21^ exclusion of marginalized groups from receiving warning information,^22^ overreliance on technical components of an EWS,^20^ limits to accuracy,^23^ and an uneven progress across different components of EWS.^17^ A detailed discussion of the current gaps in EWS is widely covered in the literature.^5^^,^^20^

## Why a people-centered approach to mhews is important

### People-centered approaches as key in MHEWS

**Figure 1:**
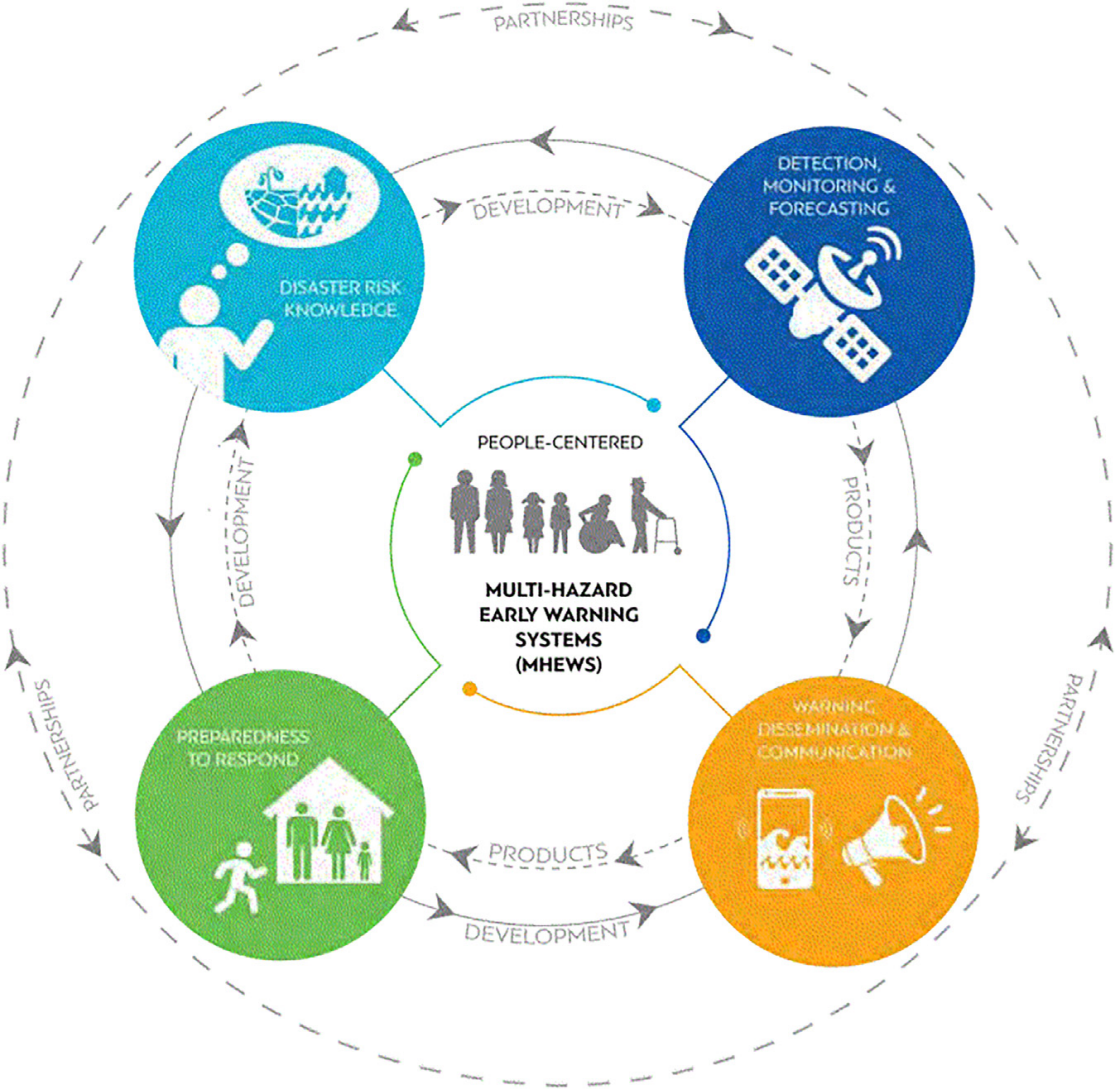
Four pillars of an MHEWS

For MHEWS to be effective, they must be “people-centered”, serving those affected by natural hazards. This concept gained traction with the Hyogo Framework for Action 2005–2015,^24^ which highlighted the need to address vulnerabilities, offer actionable advice, and assist decision-makers. This emphasis is evident in the global framework for MHEWS (Figure 1), often referred to as a framework for people-centered EWS, where people are central to the four pillars: (1) disaster risk knowledge, (2) observations, monitoring, analysis, and forecasting, (3) warning dissemination, and (4) preparedness and response capabilities.

Being people-centered means that MHEWS should involve at-risk communities, ensuring inclusivity for all community members.^18^ The goal of people-centered EWS is to enable individuals and communities at risk to take timely and appropriate actions, minimizing the risk of injury, loss of life, and harm to property and the environment.^25^ EWS become people-centered by addressing individuals’ and communities’ specific vulnerabilities and capacities, considering factors like gender, age, disability, mobility, language, and culture to ensure no one is left behind. By challenging the hazard-focused paradigm, this approach advocates for tailored warning systems that reduce vulnerabilities, while recognizing that root causes and systemic pressures shape access to resources and impact preparedness, response, and recovery from hazards.^26^

Emphasizing people-centered approaches is crucial for several reasons, especially in the Global South.

(1) Those most affected by disasters have the most to gain from disaster risk reduction (DRR), making it a local issue.^27^ The region has pioneered community-based DRR, underscoring the value of local participation in developing sustainable, context-specific solutions.^28^ For instance, in Peru, the Participatory Monitoring Network (red MoP) uses citizen science and low-cost technology for rainfall monitoring, strengthening environmental citizenship, and offering timely information that help communities take early actions.^29^
(2) At risk communities are not monolithic, but consist of individuals with varying vulnerabilities and capacities, which is crucial for creating an EWS that serves everyone. Factors such as gender, race or ethnicity, education, age, disability, LGBTQI+ status, and indigenous identity affect how people are impacted by hazards and their ability to prepare for and respond to disasters.^30^ For example, in Nepal, women preferred receiving in-person or verbal warning messages to text-based mobile phone alerts, due to gendered literacy, language, and phone ownership barriers.^31^
(3) At-risk communities possess rich traditional, local, and indigenous knowledge that informs all four components of the MHEWS pillars.^32^ For example, in Bolivia, Practical Action are working with indigenous groups to learn from their long-term understanding of risks and methods of anticipating hazards to improve the EWS for everyone, as well as improving the access to appropriate alerts to remote indigenous communities.^33^

A critical analysis of “people-centered” risk reduction approaches reveals that, while their importance is recognized, this often does not translate into practice. Many DRR initiatives, including MHEWS, remain top-down and lack meaningful inclusion of people.^26^^,^^34^ We argue that focusing on people is even more crucial in the context of multi-hazards, given their unique impacts, potential to increase risks, and the way they change the risk landscape.

### Implications of multi-hazard thinking for an EWS

As mentioned in the Introduction, the term “multi-hazards” covers both multiple single hazards in place and how these hazards are interrelated in time and space. Here, we present what we identified to be the four core concepts from the existing body of knowledge on multi-hazards that we see crucial for moving toward MHEWS.

#### Concept 1: Multi-hazard interrelationships

Since 2010, there has been a growing body of literature dealing with different classifications of how hazards can interact in time and space.^35^^,^^36^^,^^37^ In this perspective, we use the aggregated classification by Gill et al.,^38^ presented in Table 1.

**Table 1:** Types of multi-hazard interrelationships

Interrelationship type	Description	Example implication for MHEWS
Triggering	One hazard triggers one or more other hazards. For instance, rainfall-triggered landslides in Nepal.^39^	Scenario of an earthquake-triggered tsunami: people advised to leave their houses due to an earthquake, then at a higher risk of a tsunami because of being outside.
Amplification	One hazard changes environmental parameters, resulting in an increased probability of another hazard occurring.^38^ For instance, heatwaves increase the probability of wildfires in East Africa.^40^	Warning thresholds need to be updated regularly, to account for changes in hazard probabilities. For instance, after a wildfire, there is an increased risk of flooding as surface runoff increases – flood warning thresholds need to be updated with the information from other hazard types.
Compound	Hazards and their impacts coincide in time and space. They can be resulting from the same primary event or driver or have no underlying interrelationship.^38^^,^^41^ For instance, compound flooding in India caused by heavy rainfall and water level rise in Southern Kerala in India,^42^ or COVID-19, Cyclone Amphan, and monsoon floods in Bangladesh in 2020.^43^	The impact of the hazard is likely to be affected by two hazards occurring simultaneously. This has a direct impact on early action and communication of risks – e.g., whether to shelter in place (to avoid exposure to COVID-19) or to evacuate (to escape flood waters), whether to shelter in the basement (to avoid wind damage from storms or tornadoes) or on the top floor (to avoid flooding). If there are dual warning systems for different hazard types, people may be receiving conflicting alerts from different sources advising to do different early actions.
Consecutive	One or more hazards occur in succession, with their direct impacts overlapping in the same area, while recovery from the initial event is still underway.^15^ For instance, consecutive Cyclones Idai and Kenneth in Malawi, Mozambique, and Zimbabwe in 2019.	Due to losses incurred by an initial event, people’s capacities to access resources and prepare for a consecutive event are diminished. Disaster-related migration and/or displacement from the primary hazard can also place people at greater risks to subsequent hazards.

As shown in Table 1, hazards interact in various ways across time and space, making these dynamics challenging to capture. Efforts are increasing to reclassify past events to characterize the spatiotemporal footprints of multi-hazards,^44^^,^^45^ as the lack of multi-hazard databases is a major obstacle to advancing multi-hazard risk management.^14^ This task is particularly complex in the Global South where data on past events and their impacts are limited.^46^

In the context of MHEWS, it is important to note that some hazards are inherently multi-hazardous, as reflected in their warnings. For example, volcanic activities are multi-hazard events involving lava flows, gas emissions, seismic activity, lahars, and landslides.^47^ In Peru, the Instituto Geofísico del Perú monitors 12 of 16 volcanoes in the south, publishing bulletins for volcanic unrest, ash dispersion, and lahars.^48^ For tsunamis, existing warning systems mainly focus on those triggered by earthquakes, such as the Pacific Tsunami Warning System.^49^ However, there are still few operational examples of MHEWS that account for interacting hazards.^19^

#### Concept 2: Inclusion of the dynamics of vulnerability in the multi-hazard context

In a multi-hazard context, the focus is often on hazard interrelationships. However, it is crucial to recognize that vulnerability is also dynamic, meaning the needs, vulnerabilities, and capacities of at-risk people will change in multi-hazard scenarios. De Ruiter and Van Loon^50^ identify three types of vulnerability dynamics relevant to a multi-hazard context.

(1) The underlying dynamics of vulnerability: vulnerability is not constant, even in the absence of hazards, as peoples’ conditions change (e.g., income levels and access to healthcare and credit).
(2) Changes in vulnerability during long-lasting disasters: for example, people experiencing long lasting droughts will have increased vulnerabilities as the drought is progressing.
(3) Changes in vulnerability during multi-hazard scenarios: for example, people’s houses were impacted by an earthquake and then consecutively hit by a flood.

The dynamics of vulnerability often go unaccounted for in risk assessment and management^14^^,^^37^ and receive far less emphasis than hazard interrelationships. However, we argue that considering these dynamics is crucial for MHEWS, as they are vital for realistic risk assessments^50^ and understanding what early actions people can take. This is especially relevant in the Global South where disaster risk is primarily driven by high vulnerabilities rather than hazard characteristics alone^51^; therefore, vulnerabilities, social relations, and existing capacities must be considered as determinants of disasters.

#### Concept 3: Multi-hazard impacts are higher than impacts of single hazards

By identifying multi-hazard events during the past 123 years (1900–2023) using the EM-DAT database, Lee et al.^45^ find that 19% of 16,535 disaster records are classified as multi-hazard events; however, they caused 59% of global economic losses. There is a growing consensus and evidence that impacts of interrelated hazards are higher compared to single hazards alone.^15^ For example, the failure of landslide-caused river dams have caused significant damages in Nepal from their resultant outburst mega floods.^52^^,^^53^ Important to note is that in a multi-hazard scenario, it is not just interrelated hazards that result in a heightened impact, but also the dynamics of vulnerabilities of people and assets.

The lack of multi-hazard event and impact data in the Global South^54^ raises concerns about the accuracy of risk assessments used for MHEWS. This is further complicated by the cascading and indirect nature of multi-hazard impacts that are often inadequately covered in risk assessments.^37^ Boult et al.^19^ argue that considering the interacting nature of hazards would lead to more accurate impact assessments in MHEWS. However, our understanding of multi-hazard impacts is mostly based on case studies of individual events, lacking systematic analysis and classification.^55^ Additionally, impact data are rarely disaggregated by factors such as age and gender; for instance, only 11 out of 85 countries have any disaster impact data disaggregated by sex for mortality in the DesInventar database, and of those 11 countries, only 0.65% of recorded deaths were disaggregated.^56^ Yet, this information is crucial for making MHEWS “people-centered”, taking into account their differences and marginalization factors.

#### Concept 4: Thinking beyond natural hazards toward interacting risks

While this perspective focuses on natural hazards, it is important to note that these hazards often do not occur in isolation but in complex multi-risk environments. The UNDRR and International Science Council^57^ identified 302 hazards, including meteorological and hydrological, extraterrestrial, geohazards, environmental, chemical, biological, technological, and societal. As recently argued by UNDRR,^18^ including all hazards and the resulting risks is essential for MHEWS.

Considering multiple risks is especially relevant for countries in the Global South where crises often interact, compounding vulnerabilities and impacts. For example, extreme weather, environmental degradation, and socio-economic challenges at the national and regional level combine to create systemic risks in Senegal, which exacerbates food insecurity and internal displacement.^58^ Similarly, Thalheimer et al.^59^ find that extreme weather and conflicts have implications for internal displacement in Somalia, while compound events can result in systemic risks influencing food insecurity in the country.^60^ A truly people-centered approach to MHEWS requires addressing the full range of hazards and risks that people face. This means shifting from a focus on multiple natural hazards to a broader multi-risk approach that includes non-natural hazards (e.g., biological threats, food security, and conflicts) and their interrelationships.

## Integration of multi-hazard thinking across different pillars and cross-cutting components of MHEWS

Figure 1 presents the four pillars of a people-centered MHEWS, which are complemented by four overarching components^25^: (1) effective governance and institutional arrangements, (2) local community involvement, (3) a multi-hazard approach, and (4) consideration of gender perspectives and cultural diversity. For an MHEWS to be effective, all these components must be addressed.^5^ An “end-to-end” EWS should integrate these elements across sectors, linking hazard monitoring with the dissemination of vital information and guidance to protect lives, property, and livelihoods.^18^

The latest Global Status of MHEWS report^17^ highlights significant disparities and uneven progress across the four pillars, with the highest reporting for Pillar 3 on communication and dissemination and the lowest for Pillar 1 on disaster risk knowledge. The report stresses that these pillars are highly interconnected, and failing to deliver on one could compromise the entire system (*ibid.*). There are also notable regional differences; for example, the EW4All initiative found that only a third of WMO states have multi-hazard monitoring and forecasting systems, with major gaps in Africa, the Pacific, and Latin America.^61^

**Figure 2:**
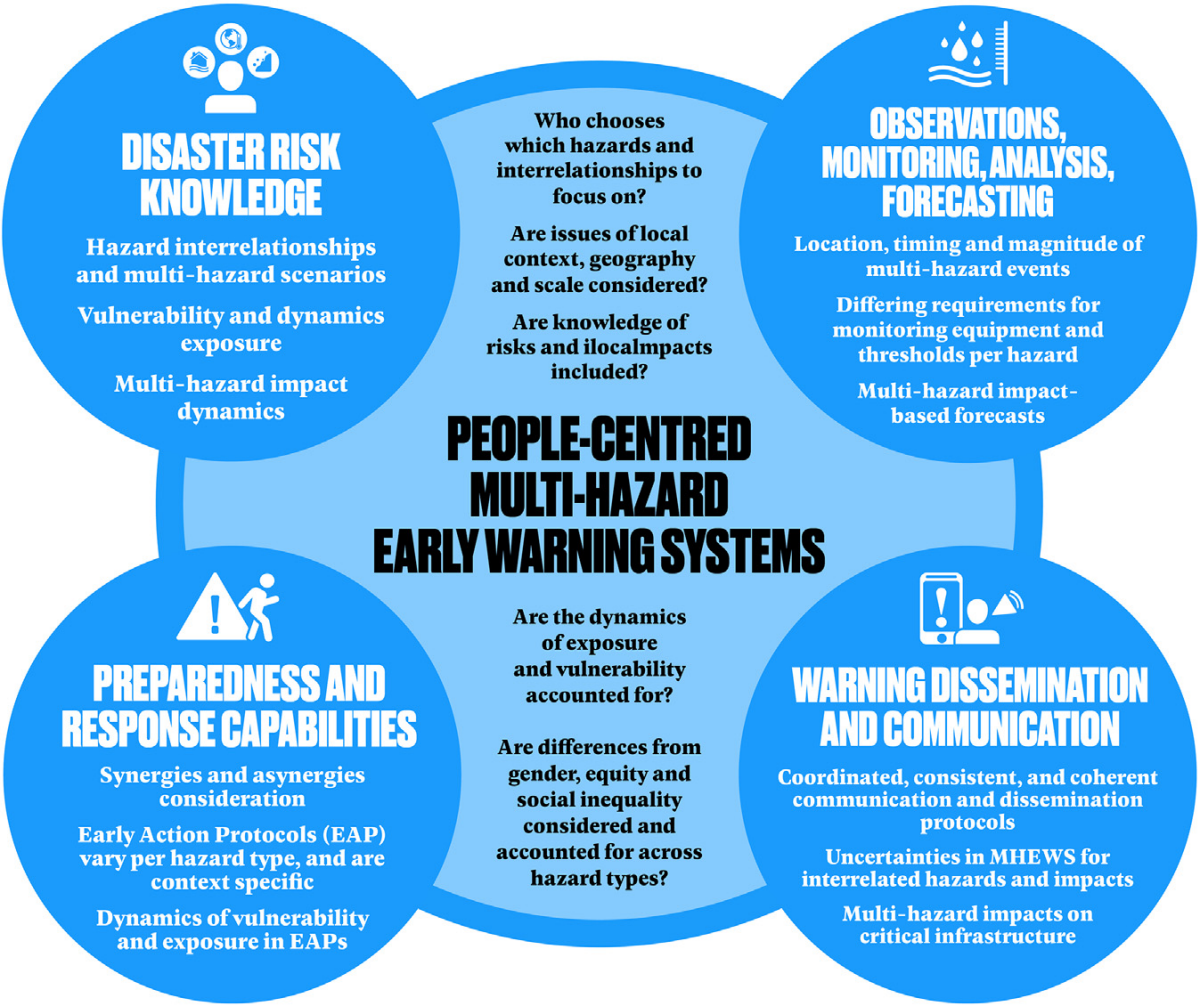
Multi-hazard considerations across four pillars of MHEWS, together with the main considerations for making MHEWS people-centered

In this section, we will reflect on what implications does multi-hazard thinking have on these different pillars of MHEWS (The cross-cutting component of multi-hazard approach will not be covered as this is the overall focus of this perspective paper.). We aim to outline key challenges and considerations for building a people-centered MHEWS. While this overview cannot cover all aspects in detail, we hope to spark further discussion and progress. A summary of the main points is in Figure 2.

### Pillar 1: Disaster risk knowledge

Disaster risk covers hazard, vulnerability, and exposure. Significant advances in multi-hazard risk assessment have been made, including qualitative, semi-quantitative, and quantitative methods.^62^ However, most approaches still treat hazards separately, without considering their interrelationships,^63^ due to challenges like hazard comparability, data, and uncertainties.^13^ Adding vulnerability dynamics increases complexity, with no standardized methods available.^50^^,^^64^ In the context of people-centered MHEWS, this means the following.

(1) *Hazard*: The full range of hazards and their interrelationships, including spatiotemporal evolution, must be considered. People at risk, especially the most vulnerable, should be central in prioritizing hazards, including non-natural ones, identifying relevant interrelationships, and setting impact thresholds.
(2) *Vulnerability*: Risk assessments must consider vulnerability dynamics, especially during consecutive events, as initial impacts can increase vulnerability to future events. They should also account for diverse vulnerabilities within a community, including factors like age, gender, and social status. The relevance of these factors varies by hazard; for instance, age affects heat wave coping,^65^ while mobility disabilities matter more for rapid-onset hazards like floods.^66^
(3) *Exposure*: Like vulnerability, people’s exposure can change during a multi-hazard event. For example, Šakić Trogrlić et al.^67^ found that in Mathare, Nairobi, people were moved to higher ground after floods, even though these areas were at risk of landslides. During the 2023 Hawaii wildfires, controversy emerged over why the signal sirens were not used. Officials explained the sirens were intended for tsunamis and might have directed people toward the hills, where wildfires were present.^68^

Risk assessment for MHEWS must address impacts of both single and interrelated hazards.^18^ To make MHEWS people-centered, it should prioritize impacts identified by locals and how they affect vulnerabilities. For instance, in Peru, cold waves impact only a few in specific areas, but those affected are among the poorest and, quite often, invisible in official geo-spatial information, making EWS crucial for them.^69^ Deciding which hazards to prioritize in MHEWS requires careful consideration.

Recently, significant efforts have been made in developing impact-based forecasting (IBF), which combines forecast information with data on vulnerability and exposure to shift from predicting “what the weather will be” to “what the weather will do”.^19^

### Pillar 2: Observation, monitoring, analysis, forecasting

Forecasting hazards depends on scientific knowledge of natural processes, historical data, and continuous monitoring.^70^ Hazard forecasts provide information on location, timing, and magnitude, but this requires diverse data sources, advanced modeling, and computational power, which differ between the Global North and South. The challenge increases with interrelated hazards.^71^ For example, Schroeter et al.^72^ note that uncertainties limit multi-hazard forecasting, while Láng-Ritter et al.^73^ highlight the difficulty of forecasting compound flooding due to different governing physical processes.

Examplesof observation, monitoring, and forecasting systems that account for multiple hazards do exist. For instance, KIKIKURU in Japan jointly monitors and forecasts rainfall-related hazards like landslides, inundation, and floods.^18^ The Copernicus Emergency Management Service continuously monitors for hazard signals and integrates systems like the Global Flood Awareness System, the Global Drought Observatory, and the Global Wildfire Information System. However, significant global gaps remain. Among the 30 countries analyzed by the EW4All initiative, most have basic or less-than-basic capacity to monitor priority hazards, relying mainly on global or regional model outputs.^17^ This raises questions about how people-centered these products are and their ability to provide locally relevant information for decision-making and early action. Forecasting interrelated hazards is still in its early stages.^5^

**Figure 3:**
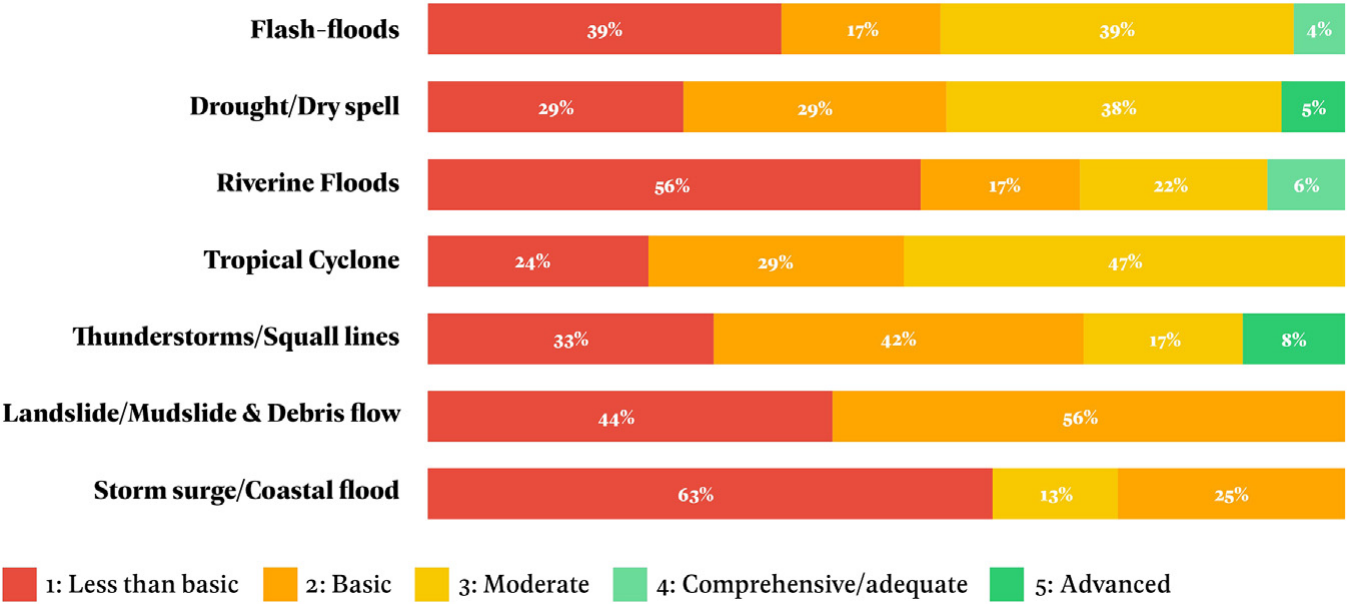
MHEWS observation and forecasting capacity level by hazard type for 30 selected EW4All countries

Forecasting different hazards requires varied technologies depending on the hazard group. For example, UNDRR^18^ notes that hydro meteorological hazards need weather stations and meteorological satellites, while geohazards rely on seismometers, sea buoys, and earth observation satellites. In many Global South countries, access to these technologies and ongoing maintenance is often limited. Monitoring needs and data availability also differ widely across hazards like floods, wildfires, earthquakes, and landslides. Local data quality is often poor; for instance, only 26% of stations in Africa met WMO standards in 2019.^74^ Additionally, monitoring and observation quality varies by hazard type (Figure 3), and just 31% of WMO members have the systems needed for monitoring and forecasting multiple hazards simultaneously or cumulatively.^17^

From a multi-hazard, people-centered perspective, information on potential impacts is crucial. IBF combines hazard forecasts with data on exposure and vulnerability, helping local communities take early action. For example, in Nepal, the Department of Hydrology and Meteorology and the UK Met Office have developed impact-based forecasts for rainfall-triggered landslides.^75^ In a multi-hazard context, IBF must account for vulnerability dynamics and the differentiated impacts to ensure safe warnings. Currently, guidelines do not specify how multi-hazard IBF should work,^19^ and our understanding of multi-hazard impacts is limited.^55^ Merz et al.^76^ argue that the next challenge is shifting from single-hazard to multi-hazard impact forecasts while considering hazard and vulnerability interactions. IBF relies on hazard magnitude thresholds as triggers for warnings,^77^ but while progress has been made in developing thresholds for hazards like floods, there is a need to establish thresholds that meet the needs of the most vulnerable and capture localized impacts of different hazards.

### Pillar 3: Warning dissemination and communication

Dissemination refers to how warnings reach end-users, while communication concerns the content of the information.^5^ This is a crucial pillar of an EWS, as failures often arise from poor communication and dissemination.^78^ Warnings must clearly convey potential impacts, how they affect the audience, and the hazard’s timing and location.^11^ In people-centered EWS, involving those at risk in the design process and tailoring warnings to account for differences in access, understanding, and ability to act is essential (e.g., people with disabilities, remote areas, migrants, tourists).^18^ For example, in Bangladesh, Practical Action works with the Flood Forecasting and Warning Centre (FFWC) and the Bangladesh Meteorological Department (BMD) to deliver voice alerts, translating warnings into easy-to-understand messages with advice on protecting assets and livelihoods. These messages are based on contextual information gathered through community work and trained local resilience agents (LRAs).^79^

In the context of multi-hazards, additional challenges arise, for instance.

(1) How to develop coordinated communication and dissemination protocols that address varying vulnerabilities to different hazards without increasing exposure to other risks.
(2) Interrelated hazards create greater uncertainties than individual hazards, and communicating these uncertainties effectively is still underexplored.^80^ For example, cascading impacts are hard to assess, leading to higher uncertainties in impact-based forecasts.
(3) Connectivity infrastructure can be impacted differently by various hazards, so redundancy should be built in to ensure warnings are communicated even if parts are damaged.

Coordinated messaging is essential in a multi-hazard context. Agencies must align communication and dissemination protocols to offer a unified source of information, preventing conflicting messages that burden people. Consistent, coherent alerts should be agreed upon in advance. For example, Bangladesh has a strong cyclone EWS with a strong network of volunteers to disseminate messages to communities at risk; Practical Action is building on this system to integrate flood early warnings in a coordinated way. Common Alerting Protocols (CAPs), as an international standard for emergency alerting and public warning, can serve as a foundation for communicating and disseminating multi-hazard warnings as they cover many hazard types: weather events, earthquakes, tsunamis, volcanoes, public health crises, power outages, and other types of emergencies.^18^ However, there are significant problems with uptake of CAPs in least developed countries where the information and communication technologies are often limited.^81^ Furthermore, additional analysis and research is needed into how CAPs can effectively account for hazard interrelationships.

### Pillar 4: Preparedness and response capabilities

From a multi-hazard perspective, it is important to consider the synergies and asynergies between actions taken for different hazards. Synergies occur when a response to one hazard also benefits another (e.g., pre-emptive evacuations from coastal areas can protect against both tsunamis and storm surges using the same routes and shelters). In contrast, asynergies arise when managing one hazard increases the risk of another (e.g., allocating water to agriculture during a drought may reduce water availability for firefighting, increasing wildfire risk).^82^

The anticipatory action movement offers a valuable framework for assessing multi-hazard preparedness and response capacities. Anticipatory action aims to reduce the humanitarian impact of a predicted hazard before it strikes,^83^ often through early action protocols (EAPs), which outline agreed actions based on specific triggers. However, EAPs are typically hazard-specific and do not fully engage with the reality of multi-hazards.^12^ It is essential to examine synergies and asynergies in multi-hazard scenarios. For example, a flood EAP may call for evacuation, but if a cold wave occurs simultaneously, blankets and clothing would also be necessary. Similarly, flood shelters could double as cooling centers during heatwaves. Vulnerability and exposure dynamics must be considered when developing EAPs to maximize synergies and minimize asynergies between response options.

Implementing preparedness and response requires considering the needs of all exposed and at-risk populations, including marginalized people or groups that have specific support needs, to improve response planning.^18^ From a multi-hazard perspective, this involves understanding which vulnerabilities are most affected by specific hazards (e.g., sanitation workers in Bangladesh are highly at risk from heat waves due to working outdoors but are often the poorest and cannot afford not to work) and how this impacts people’s ability to take action.

### Cross-cutting component 1: Effective governance and institutional arrangements

A governance framework is needed to integrate all four components of MHEWS, facilitating coordination among those responsible for each^18^ and creating a supportive environment for MHEWS implementation. However, the governance of multi-hazards is still under-researched. Recent studies in Europe, Istanbul and Nairobi, suggest that stakeholders see governance issues as major obstacles to multi-hazard risk management.^14^^,^^67^ Research shows that siloed approaches, where individual hazards are managed by separate agencies without coordination, data sharing, or clear responsibilities, could hinder MHEWS. For example, in Nepal, a mandate for EWS is assigned to the Department of Hydrology and Meteorology, who have expertise on floods and weather-related hazards, whilst landslide expertise that is essential for landslide EWS resides in the Department of Mines and Geology. Effective MHEWS require defined responsibilities, shared data, expertise, and clear cross-institutional collaboration procedures.

The UNDRR’s *Words into Action* guide^18^ covers governance for MHEWS, including legal, policy, accountability frameworks, international cooperation, technology, and financing. However, Šakić Trogrlić et al.^5^ highlight that even single-hazard EWS governance is difficult, with systems often underfunded, laws focused on response, and responsibilities spread across departments.^9^ The complexity of multiple hazards adds to this challenge, involving more institutions, stakeholders, and interoperable data. Despite this, examples like Peru’s Agroclimatic Platforms, led by the Servicio Nacional de Meteorología e Hidrología del Perú (SENAMHI) and the Ministerio de Desarrollo Agrario y Riego (MIDAGRI), show effective governance, fostering dialogue between hydro-meteorology, agriculture sectors, and local actors to support climate-smart, sustainable agriculture. From a people-centered approach, governance should actively involve those at risk in decision-making, prioritizing hazards, communication channels, and actions, ensuring that the most vulnerable are heard in the co-design of MHEWS.^31^

In terms of disaster risk governance, a shift toward people-centered MHEWS presents an opportunity to progress from addressing risks from isolated hazards toward managing interconnected, compound and complex risks, due to several reasons. Among others, these include: (1) MHEWS require cross-sectoral and cross-scalar (i.e., from local to national levels) collaboration, (2) higher emphasis on community and citizen engagement due to the “end-to-end” focus of the four-pillars framework, (3) streamlined resource allocation and optimization resulting from integrated forecasting, monitoring, dissemination, communication and response mechanisms, and (4) comprehensive risk assessments encompassing all relevant hazards and their interactions serving as a key for informing decision making. For enabling these opportunities, there is a requirement for cross-institutional government arrangements, including data sharing, collaborative working modalities, and shared strategic development plan.

### Cross-cutting component 2: Involvement of local communities

Previous sections provided a detailed rationale as to why inclusion of people at risk is a paramount for effective MHEWS. For example, the inclusive early warning system in the Rimac River basin in Peru has a community approach, addressing the different needs and primary risks that threaten the lives and livelihoods of families, increasing their capacity to understand hydrometeorological risk scenarios, recognize warning messages, and know what to do about each type of message. Early action protocols are organized through community Civil Defense brigades to support an orderly evacuation in case they are required to move to safe areas.^31^

Involvement of local communities informs all four core pillars of an MHEWS, for instance.

(1) *Disaster Risk Knowledge*: Peoples’ local knowledge serves as an entry point for understanding which hazards and hazard interrelationships to focus on, as well as what are the place-specific exposures, vulnerabilities, and impacts.
(2) *Observations, Monitoring, Analysis, Forecasting*: Citizen Science initiatives and crowdsourced data can directly feed into real-time flood forecasting, as shown by See^84^ and Annis and Nardi,^85^ while indigenous knowledge is seen as a useful resource for seasonal weather forecasting and drought prediction.^86^ Citizen science and indigenous knowledge specific to interrelated hazards can be used to fill in the gaps on multi-hazard knowledge.
(3) *Warning Dissemination and Communication*: Inclusion of community leaders and respected figures within a community in remote areas can improve information flows, and serve as back-up channels in case of infrastructure failure.^87^ These individuals could also communicate specifics of local development of multi-hazard events and their impacts, thus helping to develop dissemination strategies more applicable to the local situation.
(4) *Preparedness and Response Capabilities*: People can identify context-appropriate early actions which take into account differentiated capacities within a community in the context of different natural hazards and their interrelationships.

### Cross-cutting component 3: Consideration of gender, Equity and social Inequalities

Early warning systems often lack gender and social inequality considerations, leading to deficiencies in addressing the specific vulnerabilities and capacities of different marginalized people.^88^ By integrating gender-sensitive and/or transformative approaches, these systems can better meet the diverse needs of communities, improve response capabilities, and foster trust in warnings.^5^ In the context of MHEWS, gender and social inequality considerations are central across the four pillars, including for example.

(1) *Risk knowledge:* Critically assessing and considering how vulnerability may change depending on hazard types and their interrelationships, e.g., people with physical mobility restrictions may be at higher relative risk from flash floods and require longer lead times to evacuate, whereas people with underlying health conditions may be at greater risk from heat waves.
(2) *Monitoring and warning*: Integrating indigenous knowledge from communities of different hazards, their interrelationships and impact into forecasting capabilities, filling in data and information gaps with historical and local knowledge.
(3) *Dissemination and communication*: Translating risk information and alert content across hazards for a diversity of languages in a consistent way that captures meaning (rather than direct automated translation) so that alerts are understandable across hazards, e.g., Typhoon Haiyan’s “storm surge” was not understood by locals and resulted in many sheltering in at-risk places.^89^
(4) *Response capabilit*y: Supporting the unique needs of marginalized people in early action protocols across hazard types, e.g., prioritizing spaces in cooling centers for people with underlying health conditions during heat waves and providing blankets in flood shelters for children and elderly during cold waves.

## Reflections on the utility of MHEWS framing and a suggested way forward

### Critical reflection on the need for and current utility of MHEWS framing

In the previous section, we detailed how multi-hazard thinking affects the different pillars and components of MHEWS. We argue that to be truly “people-centered”, EWS must inherently be “multi-hazard”, as most hazard-prone areas are rarely exposed to just one type of hazard. For example, Thompson et al.^54^ found that Kathmandu faces 21 natural hazards, while Šakić Trogrlić et al.^67^ identified 19 natural hazard types in Nairobi and 23 in Istanbul. These multiple hazards and their interrelated impacts can devastate lives and livelihoods. Therefore, any risk management option, including EWS, that does not account for these interrelationships will be incomplete and ineffective. Continuing with hazard-specific approaches will fail to build true resilience for people and nations.

The increased emphasis on MHEWS is a welcome step in the right direction, but we argue that the current approach has several issues. First, the movement for multi-hazards has been largely driven by the scientific and research community, with global policy and practice offering only broad guidelines, and practical implementation remains lacking. While research is essential, there must now be a stronger focus on people and implementation. Advancements in physical sciences are promising, but under current pressures we cannot afford to wait for “perfect” science. Second, the current MHEWS framework reflects ambition rather than reality, with the two often misaligned and rarely discussed outside academic circles. The multi-hazard concept is not fully addressed, especially regarding hazard interrelationships, and the dynamics of vulnerability and exposure are largely overlooked, even though these are core risk components. This gap risks raising expectations that the current MHEWS practices cannot meet.

The global momentum from the EW4All initiative presents a crucial opportunity to assess and advance our understanding of MHEWS, clarify our goals, and outline the process to achieve them. However, we need a reality check on the ambition of moving toward MHEWS. Accurate reporting is essential to understand the true status of MHEWS, including which systems account for hazard interrelationships and the regional differences. This information will help track progress in both the Global North and South and identify areas needing more effort. Additionally, while pursuing MHEWS, we must address unresolved issues of single-hazard EWS, including coverage, effectiveness, performance, and the inclusion of social dynamics.

Given the many challenges and complexities involved, a question needs to be asked: is the consideration of various interrelationships and dynamics simply too complex, and especially in the context of the Global South, where data are limited and governmental capacities are often low? Is the ambition simply “unrealistic”? We argue there is a way forward, and in the section below, we identify some opportunities to work toward MHEWS.

### Opportunities to work toward MHEWS

The transition to MHEWS will vary depending on local conditions (e.g., the presence and functionality of single-hazard EWS). While some places are just beginning, others are more advanced. In all contexts, the systems must reflect people’s priorities regarding relevant multi-hazards, as well as dynamic vulnerabilities, exposures, and impacts.

**Figure 4:**
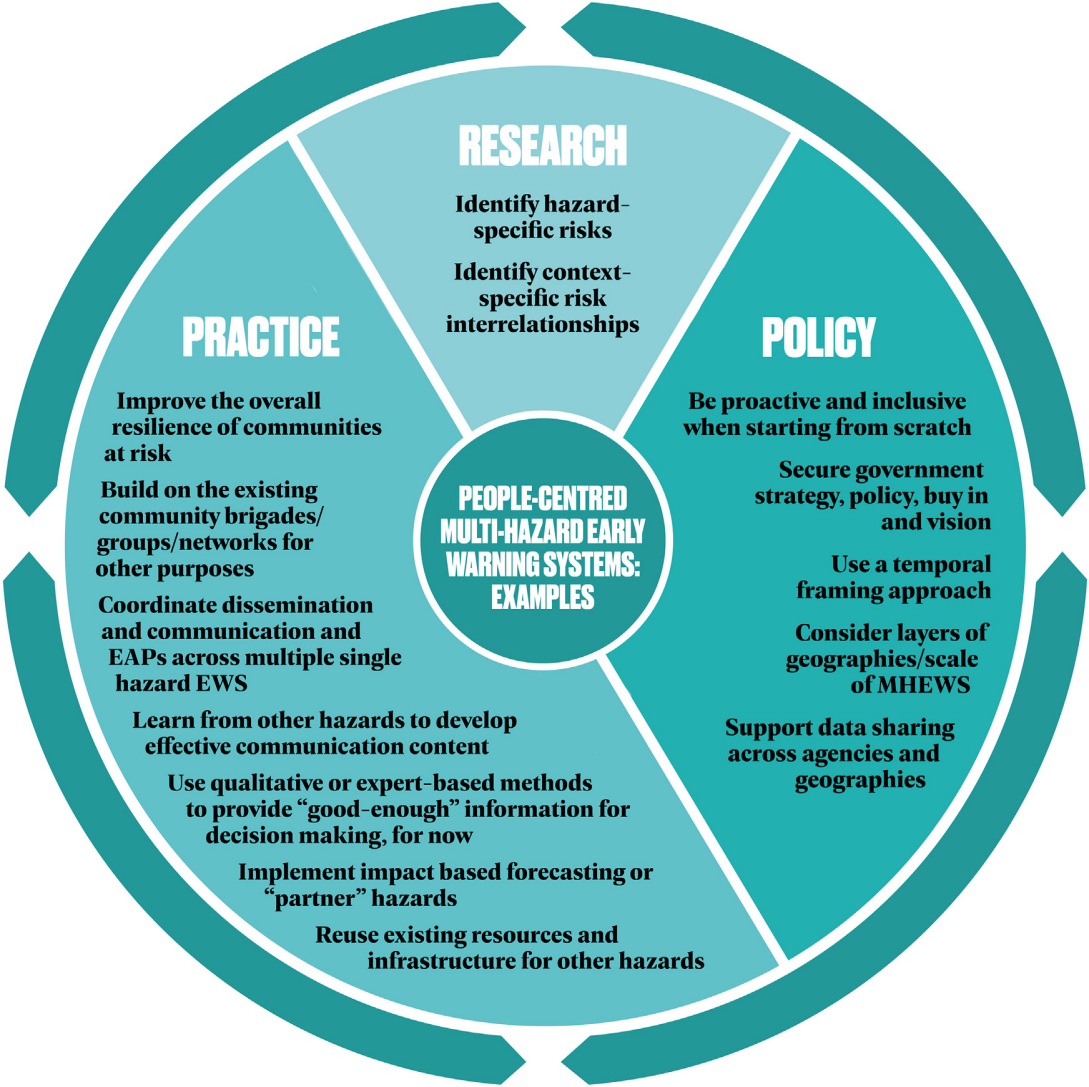
Potential entry points to work toward MHEWS, with examples from the Global South

Figure 4 outlines potential entry points for working toward MHEWS with some examples we have seen in the Global South, with Table 2 providing more detail of these examples. This is by no means an extensive list, nor a holistic conceptual framework of an MHEWS; however, we hope these examples provide some clear and concrete opportunities to begin a process of working toward MHEWS.

**Table 2:** Examples of working toward MHEWS from the Global South

Sector	Examples from Global South
Research	*Identify hazard-specific risks*: Working with communities at risk, through participatory engagement, and especially with those most vulnerable within these communities, is central to developing this understanding. Gauge whether all relevant hazards are covered by existing EWS and identify those missing. Conduct inclusive research through a range of participatory methodologies to understand differential vulnerabilities specific to each hazard type, recognizing that vulnerability is dynamic and some people are more vulnerable to some hazards compared to other hazards. Assess exposure to hazard types, including spatial and temporal changes and differences between hazards. This information can be used to inform the strategic establishment of single-hazard EWS for the priority hazards and for those most at risk.
	*Identify context-specific risk interrelationships:* Multiple single-hazard EWSs should transition toward multi-hazard EWS which account for those hazard interrelationships with the highest priority for stakeholders (identified through participatory engagement), starting for those interactions which are easier to account for (i.e., when there is an underlying physical-process dependence between hazards). Moreover, they should explore the most common threads of vulnerability and exposure dynamics in a multi-hazard scenario.
Practice	*Improve the overall resilience of communities at risk:* Community resilience can be characterized by different community capitals, including human, social, physical, financial, and natural capital.^90^ General risk reduction activities in communities, including risk mitigation and disaster preparedness activities focused on strengthening these capitals result in increased community resilience irrespective of hazard type. This can in turn result in heightened response capacity and reduced vulnerabilities, eventually having a pay out in terms of MHEWS establishment.
	*Build on the existing community brigades/groups/networks for other purposes:* Community groups and brigades that are formed for one hazard (e.g., floods) can also be used to prepare for or connect to other hazards. For instance, in Peru and Bolivia community groups managing floods are also active in response to earthquakes, wildfires, and COVID.^91^ This practice can be replicated elsewhere.
	*Coordinate dissemination and communication and EAPs across multiple single hazard EWS*: Stakeholders responsible for and included in the process across all levels (i.e., from local communities to government agencies) should work on connecting multiple single hazards EWS through coherent dissemination and communication practices and coordinated early action protocols across hazards. This would then ensure that no conflicting information is given to end users.
	*Learn from other hazards to develop effective communication content:* There is an opportunity to learn from risk communication strategies across different hazards. For instance, weather and flood alert language has improved significantly over decades, becoming more understandable and actionable. Similarly, volcano alerts inherently consider multi-hazards and uncertainties. By building on previous learning from communicating complexities and uncertainties of existing hazards for EWS, we can develop alerts and risk information that is tailored to different hazard types, and clearly articulate the complexities of multi-hazard interactions, to support effective decision making.
	*Use qualitative or expert-based methods to provide “good-enough” information for decision making, for now:* Although the dynamics of vulnerability, exposure, and impact is challenging to include, an interim approach (in the absence of “robust” scientific methodologies and while these are being developed) could be used; for instance, Boult et al.^19^ suggest real-time expert judgments as a means to account for multi-hazard dynamics. Classification of “experts” should include a range of disciplines (i.e., physical science, social science, engineering, humanities), as well as local, traditional, and indigenous knowledge.
	*Implement impact-based forecasting or “partner” hazards:* Impact-based forecasting has advanced significantly and helps communicate potential cascading impacts between hazards in a way that prioritizes relevant, understandable, and actionable information on possible impact to people at risk. Grouping hazard alerts and risk information with similar or related physical hazards or impacts can also highlight cascading or compounding effects. For example, in Bangladesh, cyclone alerts include tidal surge information, and rainfall alerts warn of possible rainfall-triggered landslides.
	*Reuse existing resources and infrastructure for other hazards:* Existing monitoring stations for monitoring one hazard could be expanded to inform monitoring of other hazards. For instance, flood monitoring stations (discharge and precipitation) that currently record water levels and temperature can be used beyond floods for heat, cold, drought, glacial lake outburst floods etc. Moreover, citizen science and local agent approaches to monitoring can support community engagement in the EWS. Similarly, flood shelters could be adapted to be cooling centers during heat waves, as well as provide a space for community activities that support longer-term resilience and development.
Policy	*Be proactive and inclusive when starting from scratch:* Given the time and resources involved, an opportunity could be used to establish a strong basis for the full multi-hazard EWS with a focus on the people-centered approach, and creating a shared vision amongst EWS stakeholders of what the overall ambition is; for instance, working through participatory engagement methods to understand and integrate local community needs, knowledge and capacities, understanding interrelationships of interest already at these stages, gauging local response capacities for different hazards, and designing communication and dissemination practices and EAPs with communities at-risk that will work across different hazards and provide diverse communities with coherent, understandable, and actionable information to take early action in the face of complex risks.
	Secure *government strategy, policy, buy in and vision*: Setting people-centered MHEWS as a national strategic goal by government authorities provides a vision for all EWS stakeholders. It can unlock funding for innovation and inclusive participation processes, clarify collective ambitions, and enhance coordination across sectors. For example, Nepal is developing an MHEWS strategy, recognizing this is a priority future direction for the country.^92^
	*Use a temporal framing approach:* Taking inspiration from work on sub-seasonal to seasonal forecasting science, a temporal approach could support decision making across the complexities of different hazard lead times.^93^ For example, structuring alert lead times and EAPs across decadal (earthquake), yearly (monsoons), seasonal (ENSO), sub-seasonal (drought), monthly (epidemics), week (heat wave), day (flood), hour (landslide), and minute (GLOFs) forecasts. This approach could provide a way of structuring local decision-making that aligns with how people are already planning and thinking (e.g., around planting and harvesting seasons).
	Consider *layers of geographies/scale of MHEWS*: In addition to national-level EWS, community-based EWS (CBEWS) in Global South countries play a significant role in overall coverage.^94^ Local governance and CBEWS can be crucial starting points where national institutional capacity is low. Therefore, one option is to develop national MHEWS for priority hazards (e.g., floods and droughts in Sub-Saharan Africa) while also capitalizing on existing and establishing new local CBEWS that address hazards more relevant locally (e.g., for slope-scale rainfall-triggered landslides in India, or cold waves in Peru). These different layers should then be connected into a fully functional system across scales.
	*Support data sharing across agencies and geographies:* Developing MOUs and formal agreements to share data across agencies, such as hydro-meteorological, geological, and health sectors, and across geographies including connecting from local to national, and across governance borders through *trans*-boundary processes, is crucial. This can facilitate developing formal agreements, processes, and usable formats is fundamental to developing risk knowledge, forecasts, and alerts that consider interactions across hazards, and complexities of scale. For example, in India, the Landslide Forecasting Center uses weather forecasts provided by the Indian National Center for Medium Range Weather Forecasting to produce rainfall-triggered landslide forecasts.^95^

Whilst there are examples of ways in which people-centered MHEWS are being progressed in the Global South, these examples are scattered and not holistic. Significant further work is needed to develop and implement people-centered MHEWS, including: (1) further analysis of existing people-centered MHEWS practices, including in the Global North; (2) a common vision of what we need to be aiming for (which this paper aims to go some way to articulating from an NGO perspective), and a detailed and agreed conceptual framework of people-centered MHEWS developed by the EWS community; (3) development of a stepwise process or roadmap to progress from where we are now, to achieve people-centered MHEWS; and (4) piloting and testing approaches to understand best practice processes to develop MHEWS (further refining and informing the MHEWS roadmap), as well as developing context and hazard-specific solutions.

## Conclusions

Globally, there is a strong push toward MHEWS, highlighted by the recent UN initiative EW4All. In this perspective paper, informed by the experiences of an International NGO (INGO) with a rich history of working on EWS in the Global South, we critically examined the current status and framing of MHEWS by identifying the core elements of multi-hazard thinking and their impact on the different pillars and components of an EWS. To the best of our knowledge, this is the first paper offering a detailed discussion of how multi-hazard and people-centered thinking informs the existing framing of MHEWS (i.e., pillars and components of an MHEWS). We argue that for MHEWS to be genuinely multi-hazard, they need to fully account for hazard interrelationships, the dynamics of vulnerability and exposure, and the specifics of multi-hazard impacts. Additionally, if EWS are to be truly people-centered, they must be inherently multi-hazard, reflecting the reality for most at-risk people, especially in the Global South.

We demonstrate that the current framing of MHEWS is largely uncritical and does not fully represent the true concept of multi-hazard, with few examples of fully operational MHEWS. Instead of abandoning the term or ambition, efforts should focus on assessing how well existing EWS represent the multi-hazard concept. This would help identify bottlenecks and areas for improvement, ultimately increasing resilience for those at risk. A step-by-step approach is needed, beginning with an assessment that clearly indicates whether an EWS is: (1) single hazard, (2) multiple-single hazards, (3) connected single hazards, or (4) a full MHEWS that considers interactions and dynamics across scales.

The task is complex and requires progress across the science-policy-practice spectrum. We briefly discuss several opportunities of working toward an MHEWS, but no single solution exists, and a full discussion is beyond this paper’s scope. This requires a transdisciplinary effort involving engineering, physical and social sciences, and humanities as well as researchers, practitioners, and policy makers, to develop an agreed conceptual framework of people-centered MHEWS, and design and test processes to work toward that common goal. The concepts, thinking, and critical reflections outlined in this perspective paper can provide an entry point for such a discussion among the wider community, to co-develop a conceptual framework and begin testing processes to make progress toward such an agreed vision. Practical implementation and learning should now be the focus.

In this perspective piece, we have focused primarily on natural hazards and associated EWS. However, as outlined in *Implications of multi-hazard thinking for an EWS*, many communities, and especially in the Global South, are subject to many overlapping crises (e.g., food security, conflict, and displacement); therefore, a further step needs to be made toward multi-risk EWS.

More tools, case studies, and evidence of impacts, benefits, and lessons are needed to scale up MHEWS, particularly in the Global South, which faces disproportionate impacts from climate and other global changes. This would provide better guidance for implementation and a reality check on what is feasible. The priority must be on those most at risk, and engaging with these challenging contexts cannot be delayed.

## Data and code availability

This paper did not use any primary data.

## Acknowledgments

This paper was funded by the Zurich Climate Resilience Alliance. The Alliance is a multi-sectoral partnership, powered by the Z Zurich Foundation, focused on enhancing resilience to climate hazards in both rural and urban communities. By implementing solutions, promoting good practice, influencing policy and facilitating systemic change, we aim to ensure that all communities facing climate hazards are able to thrive. The authors want to thank Emily Baldwin from Practical Action for the design of the figures. Finally, we thank the three anonymous reviewers for their critical feedback that significantly improved the quality of the manuscript.

## Author contributions

Conceptualization: M.B., R.S.T., C.A., and C.M.; investigation: M.B. and R.S.T.; writing – original draft: M.B. and R.S.T.; writing – review and editing: M.B., R.S.T., C.A., M.A., O.C.V., A.C., M.C.I., A.D., L.L., G.M., A.N., M.O.C., T.R., B.R., A.S., D.U., C.A., and C.M.; visualization: M.B. and R.S.T.

## Declaration of interests

The authors declare no competing interests.

## Declaration of generative AI and AI-assisted technologies in the writing process

During the preparation of this work the authors used ChatGPT 4o in order to improve readability and language of the manuscript. After using this tool/service, the authors reviewed and edited the content as needed and take full responsibility for the content of the published article.
